# High-resolution characterization of classical and non-classical HLA diversity reveals important admixture in Brazilian indigenous

**DOI:** 10.3389/fgene.2026.1948784

**Published:** 2026-10-08

**Authors:** Newton De Freitas Centurião, Lidia Cardoso Francisco, Manuel Mindlin Lafer, Roberta Sitnik, João Renato Rebello Pinho, Giselle Lima Groetares, Elena Outon Alonso, Gabriella Camerini Maciel, Margareth Afonso Torres

**Affiliations:** Hospital Israelita Albert Einstein, São Paulo, Brazil

**Keywords:** Brazil, HLA, immunogenetics, indigenous, NGS

## Abstract

Understanding linkage disequilibrium (LD) patterns involving both classical and non-classical HLA genes across diverse ethnic backgrounds provides valuable insights into genomic block inheritance, ancestry inference in admixed populations, and the genetic architecture of immune-mediated diseases. We performed high-resolution typing of classical and non-classical HLA genes using next-generation sequencing in 80 Brazilian Indigenous from 24 families belonging to 24 distinct geographical indigenous communities. Gene copy number analysis was conducted in hemizygous and homozygous samples to confirm zygosity. Allelic and haplotypic frequencies, as well as Hardy–Weinberg equilibrium, were assessed in 50 unrelated individuals, while haplotypes were manually inferred through familial segregation analysis in the remaining families. Extensive allelic and haplotypic diversity was observed, with limited sharing across families and several haplotypes not previously described in the Brazilian population. In addition, haplotypes commonly reported in other ethnic groups were identified, likely reflecting the high degree of genetic admixture in Brazil. These findings contribute to the inclusion of Brazilian Indigenous populations in high-resolution immunogenetic studies and may have implications for ancestry inference, disease association analyses, and donor selection strategies in transplantation.

## 1 Introduction

Consistent with their critical immunological function, HLA genes represent one of the most polymorphic regions of the human genome. This extensive allelic diversity presents a markedly heterogeneous global distribution, with some alleles restricted to specific populations and others shared worldwide (Meyer et al., 2018)) The contemporary Brazilian population is the result of complex admixture events involving indigenous peoples, European colonizers, and Africans forcibly transported through the transatlantic slave trade. This unique demographic history has shaped a distinctive HLA allelic landscape that remains largely underrepresented in international databases (Pena et al., 2020; Nunes et al., 2025; Boquett et al., 2020; Halagan et al., 2018).

The HLA system is classically divided into classical and non-classical genes. Classical HLA class I (HLA-A, HLA-B, and HLA-C) and class II (HLA-DR, -DQ, and -DP) genes are among the most polymorphic loci in the human genome, and their roles in antigen presentation, disease association, and transplantation outcomes are well established. In contrast, non-classical class I genes (HLA-E, HLA-F, and HLA-G), as well as the pseudogene HLA-H, have been far less explored (Shiina et al., 2009; Kochan et al., 2013) These non-classical genes exhibit a more conserved peptide-binding groove and limited coding polymorphism compared to their classical counterparts, in addition to restricted tissue expression patterns. Such features suggest immunomodulatory and tolerogenic roles, with growing evidence supporting their influence on disease phenotypes and transplant outcomes (Carlini et al., 2016).

Understanding linkage disequilibrium (LD) patterns involving both classical and non-classical HLA genes across diverse ethnic backgrounds provides valuable insights into genomic block inheritance, ancestry inference in admixed populations, and the genetic architecture of immune-mediated diseases. Moreover, detailed haplotypic characterization may improve unrelated donor selection strategies, particularly in the context of hematopoietic stem cell transplantation, where extended HLA matching is increasingly relevant (Creary et al., 2021).

In this study, we performed high-resolution classical and non-classical HLA typing in Brazilian indigenous families using next-generation sequencing (NGS) technology. Our aim was to identify and characterize alleles and haplotypes that are specific or enriched in this underrepresented population, thereby contributing to a more comprehensive understanding of HLA diversity and its implications for immunogenetics, ancestry studies and transplantation medicine.

## 2 Materials and methods

### 2.1 Population characterization

Peripheral blood samples were collected from 80 indigenous volunteers recruited at the UNIFESP Native American Outpatient Clinic between 4 October 2018, and 19 February 2020. A detailed description of the blood collection procedures has been previously reported in Lafer et al. (2022) High-resolution HLA typing was performed at the Histocompatibility Department of Hospital Israelita Albert Einstein. The inclusion criteria comprised Brazilian Indigenous patients or accompanying family members attending the UNIFESP Native American Outpatient Clinic, representing diverse family relationships. Indigenous status was defined based on self-reported residence in Indigenous territories officially recognized by the Brazilian government, without the use of genetic markers for ethnic classification. Individuals with a history of autoimmune diseases were excluded from the study. The study protocol was approved by the institutional Research Ethics Committee (CAAE 73692717.1.3001.0071), and all participants provided written informed consent prior to enrollment.

## 3 HLA sequencing

For HLA typing, genomic DNA was extracted from whole blood samples using the QIA symphony DNA Mini Kit (QIAGEN, Hilden, Germany), according to the manufacturer’s instructions. Genotyping of the HLA-A, HLA-B, HLA-C, HLA-E, HLA-F, HLA-G, HLA-H, HLA-DRB1, HLA-DRB3, HLA-DRB4, HLA-DRB5, HLA-DQA1, HLA-DQB1, HLA-DPA1, and HLA-DPB1 loci was performed using next-generation sequencing (NGS) with the AlloSeq Tx 17 kit (CareDx, Inc., South San Francisco, CA, United States of America). This platform is based on hybrid capture technology, enabling library preparation through a simplified laboratory workflow. Sequence data analysis and HLA genotype assignment were conducted using AlloSeq Assign software, version 1.0.2 (CareDx, Inc.), with alignment to the IPD-IMGT/HLA database, version 3.45. The following parameters were considered acceptable for NGS run quality: a median cluster density of 693 clusters/mm^2^, ≥75% of bases with a quality score above Q30, ≥70% of clusters passing filter, and an error rate of ≤1%. Sample quality was considered acceptable when >95% of loci achieved a sequencing coverage depth of ≥100× and allelic balance was approximately 50%.

## 4 Data analysis

Gene copy number was evaluated in all hemizygous and homozygous samples using the Assign Copy Number tool® (CareDx), a software that assesses the number of sequencing reads allocated per sample and per locus relative to expected mean values, allowing the exclusion of allelic dropout in these samples. Mean copy number values above 1.7 were considered indicative of two gene copies in homozygous samples, whereas values between 0.87 and 1.1 confirmed the presence of a single copy in hemizygous samples.

Allelic and haplotypic frequency estimations, as well as tests for Hardy–Weinberg equilibrium (HWE), were performed using PyPop software version 1.4.1 (Lancaster et al., 2007) Only unrelated individuals were included in allele-frequency, Hardy–Weinberg equilibrium, and Expectation Maximization-based population analyses. Because the cohort comprised individuals from distinct Indigenous communities, HWE results were interpreted descriptively and not as evidence of a single panmictic population. In the 24 families in which offspring were genotyped, classical and non-classical haplotypes were manually inferred through familial segregation analysis and the classical ones were subsequently compared with the haplotypes inferred by the software to support the results, following the approach described by Osoegawa et al. (2019a).

## 5 Results

Of the 81 recruited volunteers, one was excluded from the analysis due to sample collection failure. The remaining 80 individuals were organized into 24 families belonging to 24 distinct indigenous communities, as illustrated in Table 1. Figure 1 depicts the geographical distribution of the ethnicities represented in the study.

**TABLE 1:** Distribution of study population in 24 different ethnicities.

Tribes	Number of individuals	Tribes	Number of individuals
Macuxi	4	Kaimbé	10
Guarani	10	Trumai	1
Pankararé	4	Krenak	1
Baré	2	Pankararé	4
Kaiabi	3	Mehinako	2
Kuikuro	4	Yawalapiti	1
Kayapo	11	Fulniô	2
Guajajara	2	Nahukwá	2
Pataxó	1	Bororo	2
Kamaiurá	1	Wapixana	1
Panara	4	Wauja	1
Xavante	5	Xucuru	2

**FIGURE 1:**
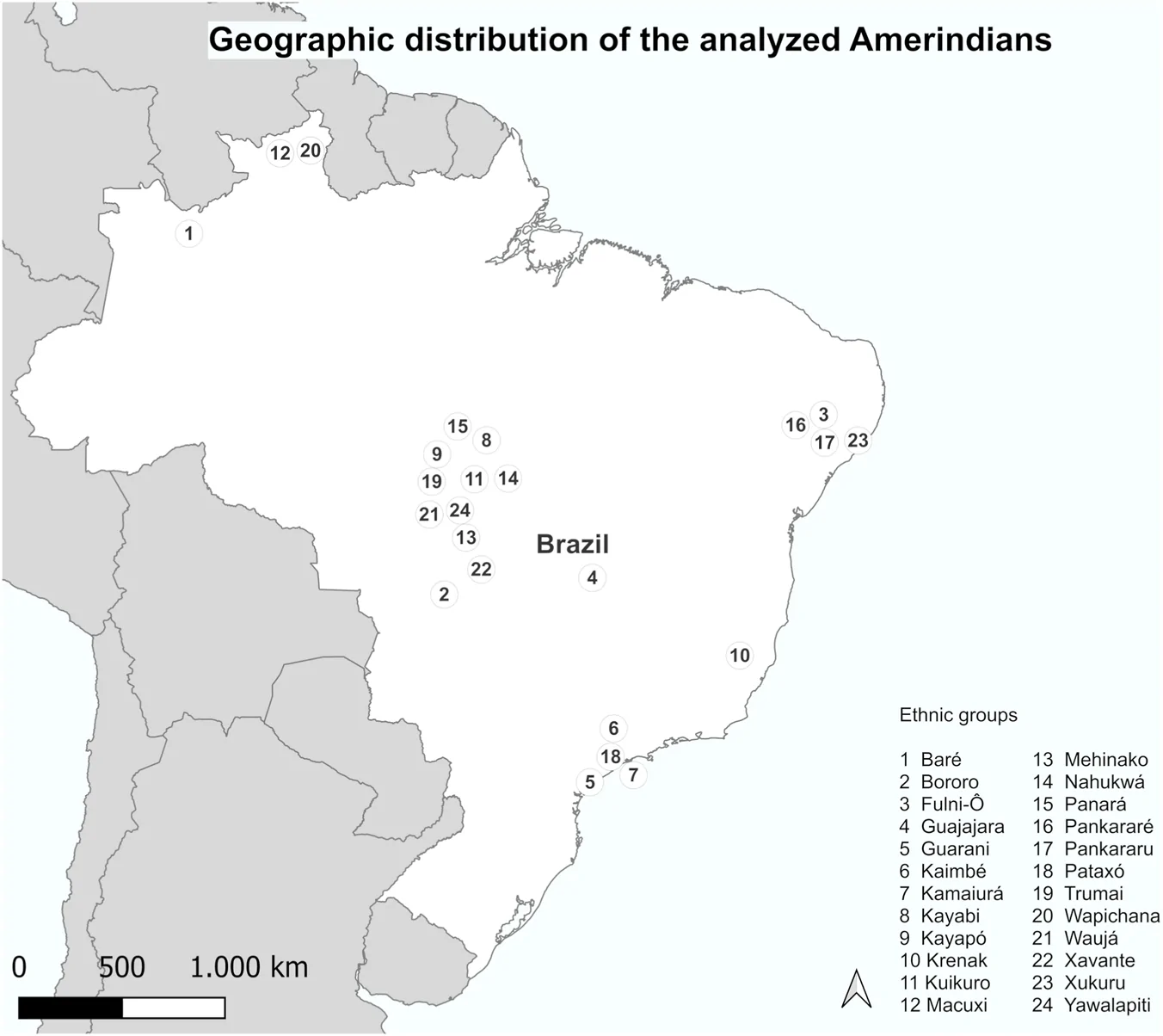
Map of the geographic distribution of the some ethnicities covered by the study.

Familial segregation analysis identified a total of 69 distinct HLA haplotypes, of which only three were shared by more than one family (Table 2), indicating a high degree of haplotypic diversity and limited haplotype sharing among families.

**TABLE 2:** Haplotypes shared by more than one family in order of frequency by manual familiar segregation. ^1^Number of families with shared haplotype. ^2^Number of individuals with shared haplotype.

HLA- F*∼G*∼H*∼A*∼ E*∼ B*∼C*∼ DRB1*∼DQA1*∼DQB1*∼DPA1*∼DPB1*	Families^1^	Individuals^2^
F*01:01:01∼G*01:01:01∼ H*01:01:01∼ A*02:01:01∼ E*01:01:01∼ B*35:05:01∼C*04:01:01∼DRB1*16:02:01∼DRB5*02:02:01∼DQA1*05:05:01∼DQB1*03:01:01∼ DPA1*01:03:01∼ DPB1*04:02:01	3	7
F*01:01:02∼G*01:01:02∼ H*02:05:01∼A*68:01:01∼ E*01:03:02∼ B*40:04:01∼C*03:04:01∼DRB1*04:11:01∼DRB4*01:03:01∼DQA1*03:01:01∼DQB1*03:02:01∼DPA1*02:01:01∼ DPB1*14:01:01	2	2
F* 01:01:01∼G* 01:03:01∼ H* 02:09∼A*31:01:02∼ E*01:03:01∼ B*35:04:01 ∼C*04:01:01∼ DRB1*08:07∼DQA1*04:01:01∼DQB1*04:02:01∼DPA1*01:03:01 ∼DPB1*04:02:01	2	2

Analysis of classical and non-classical HLA loci revealed a total of 22 HLA-A, 36 HLA-B, 17 HLA-C, 24 HLA-DRB1, 10 HLA-DQB1 and 17 HLA-DPB1 alleles. HWE tests were performed in the subset of 50 unrelated indigenous individuals and demonstrated significant deviations for all loci except HLA-C (p = 0.3468) and HLA-DPA1 (p = 05,644), probably due to reduced sample size and geographical distances between individuals. The most frequent alleles (frequency >1%) are shown in Supplementary Table S1.

Based on classical HLA genotypes, haplotypes were estimated, and those with frequencies above 1% are presented in Table 3. All estimated haplotypes were confirmed through manual familial segregation analysis. Linkage disequilibrium (LD) between HLA-B and HLA-C, as well as between HLA-DRB1 and HLA-DQB1, are summarized in Table 4.

**TABLE 3:** Observed frequency of the HLA-A*∼B*∼C*∼DRB1*∼DQB1*∼DPB1* haplotypes above 1% estimated by Expectation maximization algorithm.

HLA-A*∼B*∼C*∼DRB1*∼DQB1*∼DPB1*	Frequency
A*02:01:01∼B*35:05:01∼C*04:01:01∼DRB1*16:02:01∼DQB1*03:01:01∼DPB1*04:02:01	0.06
A*68:01:02∼B*40:04:01∼C*03:04:01∼DRB1*04:11:01∼DQB1*03:02:01∼DPB1*14:01:01	0.05
A*31:01:02∼B*35:04:01∼C*04:01:01∼DRB1*08:07∼DQB1*04:02:01∼DPB1*04:02:01	0.03
A*31:01:02∼B*39:09:01∼C*07:02:01∼DRB1*08:02:01∼DQB1*04:02:01∼DPB1*04:02:01	0.03
A*24:03:01∼B*35:04:01∼C*04:01:01∼DRB1*08:04:01∼DQB1*04:02:01∼DPB1*14:01:01	0.02
A*31:01:02∼B*39:03:01∼C*07:02:01∼DRB1*08:07∼DQB1*04:02:01∼DPB1*04:02:01	0.02
A*24:02:01∼B*35:05:01∼C*04:01:01∼DRB1*16:02:01∼DQB1*03:01:01∼DPB1*04:02:01	0.02
A*24:02:01∼B*40:02:01∼C*03:04:01∼DRB1*08:07∼DQB1*04:02:01∼DPB1*04:02:01	0.02
A*24:02:01∼B*39:05:01∼C*07:02:01∼DRB1*09:01:02∼DQB1*03:03:02∼DPB1*14:01:01	0.02
A*02:01:01∼B*15:04:01∼C*03:03:01∼DRB1*16:02:01∼DQB1*03:01:01∼DPB1*04:01:01	0.02
A*11:01:01∼B*07:02:01∼C*07:02:01∼DRB1*15:01:01∼DQB1*06:02:01∼DPB1*04:01:01	0.02
A*24:02:01∼B*39:03:01∼C*07:02:01∼DRB1*04:04:01∼DQB1*03:02:01∼DPB1*03:01:01	0.02
A*02:01:01∼B*15:04:01∼C*03:03:01∼DRB1*16:02:01∼DQB1*03:01:01∼DPB1*04:02:01	0.02

**TABLE 4:** Observed frequency of HLA-B*∼C* and HLA-DRB1*∼DQB1* haplotypes estimated by Expectation maximization algorithm.

HLA-B*∼C*	Frequency	HLA-DRB1*∼DQB1*	Frequency
B*35:04:01∼C*04:01:01	0.11	DRB1*16:02:01∼DQB1*03:01:01	0.18
B*35:05:01∼C*04:01:01	0.10	DRB1*04:11:01∼DQB1*03:02:01	0.11
B*15:04:01∼C*03:03:01	0.07	DRB1*08:07∼DQB1*04:02:01	0.10
B*40:02:01∼C*03:04:01	0.07	DRB1*08:02:01∼DQB1*04:02:01	0.07
B*40:04:01∼C*03:04:01	0.06	DRB1*09:01:02∼DQB1*03:03:02	0.06
B*39:05:01∼C*07:02:01	0.06	DRB1*14:02:01∼DQB1*03:01:01	0.06
B*18:01:01∼C*12:03:01	0.04	DRB1*03:01:01∼DQB1*02:01:01	0.05
B*39:09:01∼C*07:02:01	0.04	DRB1*08:04:01∼DQB1*04:02:01	0.04
B*39:03:01∼C*07:02:01	0.04	DRB1*04:04:01∼DQB1*03:02:01	0.04
B*15:10:01∼C*04:01:01	0.03	DRB1*15:01:01∼DQB1*06:02:01	0.04
B*07:02:01∼C*07:02:01	0.03	DRB1*01:02:01∼DQB1*05:01:01	0.03
B*18:01:01∼C*05:01:01	0.02	DRB1*07:01:01∼DQB1*02:02:01	0.03
B*15:04:04∼C*03:03:01	0.02	DRB1*10:01:01∼DQB1*05:01:01	0.02
B*53:01:01∼C*04:01:01	0.02	DRB1*01:03:01∼DQB1*05:01:01	0.02
​	​	DRB1*04:07:03∼DQB1*03:02:01	0.02

Regarding non-classical HLA genes, 24 samples (30% of the total cohort) were identified as hemizygous for HLA-H, as confirmed by the Assign Copy Number tool. Among these samples, 87.5% were in LD with HLA-A24% and 12.5% with HLA-A23. Notably, all hemizygous HLA-H samples were associated with the HLA-G*01:04:01 allele.

Due to the limited polymorphism of non-classical class I genes, a restricted number of alleles was observed: five HLA-E (E* 01:01:01, E*01:03:01, E*01:03:02, E*01:03:05 and E*01:05), four HLA-F (F*01:01:01, F*01:01:02, F*01:03:01 and F*01:NEW), ten HLA-G (G*01:01:01, G*01:01:02, G*01:01:03, G*01:01:19, G*01:03:01, G*01:04:01, G*01:04:04, G*01:05N, G*01:08:02 and G*01:NEW), and fifteen HLA-H alleles (H*01:01:01, H* 01:01:02, H*01:02:01, H*01:03:01, H*02:01:01, H*02:02:01, H*02:02:02, H*02:03:02, H*02:04:01, H*02:05:01, H*02:07:01, H*02:08:01, H*02:09, H*02:10:01, H*02:12 e H*01:NEW). The most frequent alleles (frequency >1%) and HWE tests are shown in Supplementary Table S2. The most frequent LD associations between non-classical HLA genes and HLA-A observed in the indigenous families analyzed are presented in Table 5.

**TABLE 5:** Manual haplotype segregation by familiar analysis compared to haplotype observed frequency estimated by Expectation maximization algorithm of the most frequent non-classical HLA class I genes with classical HLA-A in different families.

Locus A*	Locus E*	Locus F*	Locus G*	Locus H*	Frequency	Number of families
02:01:01	01:01:01	01:01:01	01:01:01	01:01:01	0.17	7
68:01:02	01:03:02	01:01:02	01:01:02	02:05:01	0.02	4
31:01:02	01:03:01	01:01:01	01:03:01	02:09	0.07	3
31:01:02	01:01:01	01:01:01	01:03:01	02:09	0.05	3
24:02:01	01:01:01	01:01:01	01:04:01	​	0.08	3
68:01:02	01:01:01	01:01:02	01:01:02	02:05:01	0.08	2
25:01:01	01:01:01	01:01:01	01:01:02	01:02:01	0.03	2

Four novel HLA polymorphisms were identified in this study. These included one non-synonymous mutation in exon 4 (codon 269.1) of HLA-H*01; two independent non-synonymous mutations in exon 5 (codon 276.2) of HLA-G*01 detected in unrelated individuals; and one synonymous mutation in exon 2 (codon 67.3) of HLA-F*01 identified in six samples belonging to five different families, in LD with HLA-E01:01:01. Additionally, a non-synonymous mutation in exon 3 of HLA-C*15 was detected and subsequently submitted to the IPD-IMGT/HLA database, where it was assigned the official allele designation HLA-C*15:217 (ID 62160).

## 6 Discussion

This study performed high-resolution HLA typing of both classical and non-classical genes in a sample of 80 Brazilian unrelated individuous from different Indigenous ethnicities. The widespread use of next-generation sequencing (NGS) platforms, combined with kits capable of simultaneously typing classical and non-classical HLA genes with high accuracy and throughput, enables a comprehensive assessment of the HLA region. This approach has direct applicability in population genetics studies and in the evaluation of novel factors involved in donor selection (Arrieta-Bolaños et al., 2023; Arrieta-Bolaños et al., 2012).

According to the 2022 Brazilian Census (Pochmann et al., 2020), Brazil is home to 391 Indigenous ethnic groups, totaling approximately 1,694,836 individuals (0.83% of the Brazilian population). Of these, 46.03% live in rural areas and 53.97% reside in Indigenous Lands officially recognized by the state and distributed throughout the national territory. Our dataset comprises individuals from 24 ethnicities originating from geographically distant regions, with distinct migration histories and environmental evolutionary pressures, which may help explain the low proportion of haplotypes shared between families.

Da Silva et al. analyzed high-resolution HLA typing data from approximately 298,000 unrelated hematopoietic stem cell donors and established the Brazilian catalogue of Common and Well-Documented (CWD) HLA alleles, providing a key reference for allele frequency distribution in this population (da Silva et al., 2025)However, only 0.1% of their dataset included individuals of self-reported Native American ancestry, underscoring the marked underrepresentation of this population even in national databases. This limitation is particularly relevant in the context of Brazil’s highly admixed population and may obscure ancestry-specific genetic contributions.

Most of the individual HLA alleles observed in this study are included in both the global CIWD 3.0 catalog(encompassing data from 20 bone marrow donor registries across different countries) (Hurley et al., 2020) and the Brazilian CIWD reference dataset. However, the relevance of the findings lies in the specific haplotypic combinations identified rather than in the individual alleles themselves. The occurrence of haplotypes previously reported in Indigenous populations of the Americas supports the retention of Indigenous genetic ancestry within this admixed population, despite the broad distribution of many of the constituent alleles in other populations worldwide. In this context, HLA- B is widely recognized as a robust ethnic marker due to its remarkable degree of polymorphism (Prugnolle et al., 2005; Single et al., 2020). Consistent with both CWD catalogue, the most frequent HLA-B alleles identified in our cohort (HLA-B*35:05, -B*35:04, -B*15:04, and–B*39:05) are classified as common; however, they also demonstrate a strong association with Native American ancestral lineages. These findings reinforce both the relevance of the Brazilian CWD framework and the importance of better representing underrepresented populations to improve the accuracy of genetic and clinical inferences.

HLA-DRB1 is the most polymorphic HLA class II gene. In our sample, however, most of the frequent DRB1 alleles were not specific to Brazilian Native American ancestry, apart from HLA-DRB1*08:07, which is described as common among Hispanic populations in the CIWD 3.0 catalog (Arnaiz-Villena et al., 2010). As expected, given the intense admixture that characterizes the Brazilian population, alleles commonly associated with African and European ancestries were also observed.

The three most common haplotypes A*68:01:02∼B*40:04:01∼C*03:04:01∼DRB1*04:11:01∼DQB1*03:02:01∼DPB1*14:01:01; A*02:01:01∼B*35:05:01∼C*04:01:01∼DRB1*16:02:01∼DQB1*03:01:01∼DPB1*04:02:01; and A*31:01:02∼B*35:04:01∼C*04:01:01∼DRB1*08:07∼DQB1*04:02:01∼DPB1*04:02:01 —have not been described in previous studies evaluating indigenous individuals populations (Gonzalez-Galarza et al., 2019), and only the last haplotype has a single description in allele frequency databases (Arnaiz-Villena et al., 2005). Nevertheless, patterns of linkage disequilibrium (LD) between HLA-A and HLA-B or between HLA-DRB1 and HLA-DQB1 observed in our sample have also been described in Native American populations from other countries, supporting the hypothesis of a shared ancestral branch with subsequent haplotypic recombination (Arnaiz-Villena et al., 2009; Moscoso et al., 2006).

Castro et al. published the first study evaluating both classical and non-classical HLA genes in 108 unrelated Brazilian individuals with predominantly European ancestry from Curitiba, Brazil (Castro et al., 2019) As expected, the most frequent HLA class I haplotypes including non-classical genes differed from those observed in our cohort, reflecting distinct ethnic backgrounds. However, the LD patterns involving HLA-A*02∼F*01:01:01∼G*01:01:01∼H*01:01:01 and HLA-A*24∼F*01:01:01G*01:04:01∼H*del identified in our study have also been reported in voluntary blood donors from southeastern France (Carlini et al., 2016). We identified HLA-G alleles G*01:01:19 and G*01:08:02, which are uncommon in the 1000 Genomes Project dataset (Castelli et al., 2014). Only HLA-F*01:01 and HLA-F*01:03 were observed in our cohort, consistent with findings from a Brazilian study of 199 bone marrow donors from Curitiba, where these were the two most frequent alleles (Manvaile et al., 2014). In agreement with Veiga-Castelli et al. (Veiga-Castelli et al., 2012), HLA-E*01:01:01 was the most frequent allele in our sample, followed by HLA-E*01:03:02 and HLA-E*01:03:01. Additionally, we detected HLA-E*01:03:05 and HLA-E*01:05, which were reported as less frequent alleles in other Brazilian populations by Ramalho et al. (Ramalho et al., 2017).

It is important to note that all inferred haplotypes were manually supported by family segregation analysis. However, most families consisted of only one parent and one proband, which reduces the reliability of haplotype inference compared to the gold standard configuration of both parents and two siblings (Ramalho et al., 2017; Osoegawa et al., 2019b; Askar et al., 2019). An additional limitation of this study is the potential sampling bias arising from participant recruitment at a single referral center, the UNIFESP Native American Outpatient Clinic. Although this clinic serves Indigenous individuals from multiple ethnic groups and geographically distinct regions of Brazil, the study population may not fully represent the extensive genetic diversity of the more than 390 Indigenous ethnic groups currently recognized in the country. Furthermore, geographical dispersion, restricted access to remote Indigenous territories, and logistical challenges associated with sample collection contribute to the difficulty of obtaining larger and more representative cohorts. Therefore, caution should be exercised when extrapolating these findings to all Brazilian Indigenous populations. Nevertheless, it represents the first study to characterize non-classical HLA genes and their associations with classical HLA class I genes in Brazilian Indigenous populations and the inclusion of 24 Indigenous ethnicities provides a valuable first overview of classical and non-classical HLA diversity in this underrepresented population.

## 7 Conclusion

This study provides a high-resolution characterization of classical and non-classical HLA diversity in Brazilian individuals from multiple Indigenous communities using next-generation sequencing, revealing extensive allelic and haplotypic diversity with minimal sharing between families. These findings have relevance by incorporating indigenous populations into high-resolution immunogenetic studies with potential impact on ancestry inference, disease association analyses, and donor selection strategies for transplantation.

## Data Availability

The datasets generated and analyzed in this study cannot be made publicly available due to ethical and privacy restrictions related to genomic data from Indigenous populations and the conditions established in the informed consent and ethics committee approval. Access to de-identified data may be considered upon reasonable request to the corresponding author, subject to compliance with applicable institutional and ethical requirements for the protection of Indigenous participants and their genomic data.
