# Supplementary material for: High-resolution characterization of classical and non-classical HLA diversity reveals important admixture in Brazilian indigenous
Source: Front Genet. 2026 Oct 8;17:1948784. doi: 10.3389/fgene.2026.1948784 (PMC13687286; doi:10.3389/fgene.2026.1948784)
Supplement: Supplementary file 1 [file Table1.docx]

**Supplementary Material**

| HLA-A | | | | | HLA-B | | | | HLA-C | | | | | |
| --- | --- | --- | --- | --- | --- | --- | --- | --- | --- | --- | --- | --- | --- | --- |
| Alelle | | Frequency | | | Alelle | | Frequency | | Alelle | | | Frequency | | |
| A*02:01:01 | | 0.19 | | | B*35:05:01 | | 0.12 | | C*04:01:01 | | | 0.30 | | |
| A*31:01:02 | | 0.19 | | | B*35:04:01 | | 0.11 | | C*07:02:01 | | | 0.20 | | |
| A*24:02:01 | | 0.14 | | | B*15:04:01 | | 0.07 | | C*03:04:01 | | | 0.19 | | |
| A*68:01:02 | | 0.13 | | | B*39:05:01 | | 0.07 | | C*03:03:01 | | | 0.09 | | |
| A*02:11:01 | | 0.08 | | | B*40:02:01 | | 0.07 | | C*12:03:01 | | | 0.04 | | |
| A*11:01:01 | | 0.03 | | | B*18:01:01 | | 0.06 | | C*06:02:01 | | | 0.03 | | |
| A*24:03:01 | | 0.03 | | | B*40:04:01 | | 0.06 | | C*01:02:01 | | | 0.02 | | |
| A*25:01:01 | | 0.03 | | | B*39:03:01 | | 0.04 | | C*03:04:02 | | | 0.02 | | |
| A*29:02:01 | | 0.03 | | | B*39:09:01 | | 0.04 | | C*05:01:01 | | | 0.02 | | |
| A*23:01:01 | | 0.02 | | | B*07:02:01 | | 0.03 | | C*16:01:01 | | | 0.02 | | |
| A*30:01:01 | | 0.02 | | | B*15:10:01 | | 0.03 | |  | | |  |  |  |
|  | |  | | | B*51:01:01 | | 0.03 | |  | | |  | | |
|  | |  | | | B*15:04:04 | | 0.02 | |  | | |  | | |
|  | |  | | | B*52:01:02 | | 0.02 | |  | | |  | | |
|  | |  | | | B*53:01:01 | | 0.02 | |  | | |  | | |
| HLA-DRB1 | | | | HLA-DQB1 | | | | HLA-DPB1 | | | | | | |
| Alelle | | Frequency | | Alelle | | | Frequency | Alelle | | | Frequency | | | |
| DRB1*16:02:01 | | 0.18 | | DQB1*03:01:01 | | | 0.29 | DPB1*04:02:01 | | | 0.45 | | | |
| DRB1*04:11:01 | | 0.11 | | DQB1*04:02:01 | | | 0.22 | DPB1*14:01:01 | | | 0.13 | | | |
| DRB1*08:07 | | 0.10 | | DQB1*03:02:01 | | | 0.18 | DPB1*04:01:01 | | | 0.12 | | | |
| DRB1*08:02:01 | | 0.07 | | DQB1*05:01:01 | | | 0.08 | DPB1*03:01:01 | | | 0.10 | | | |
| DRB1*14:02:01 | | 0.07 | | DQB1*03:03:02 | | | 0.07 | DPB1*02:01:02 | | | 0.05 | | | |
| DRB1*09:01:02 | | 0.06 | | DQB1*02:01:01 | | | 0.05 | DPB1*17:01:01 | | | 0.03 | | | |
| DRB1*08:04:01 | | 0.06 | | DQB1*06:02:01 | | | 0.05 | DPB1*01:01:01 | | | 0.02 | | | |
| DRB1*03:01:01 | | 0.05 | | DQB1*02:02:01 | | | 0.03 |  | | |  | | | |
| DRB1*04:04:01 | | 0.04 | | DQB1*05:03:01 | | | 0.02 |  | | |  | | | |
| DRB1*07:01:01 | | 0.04 | |  | | |  |  | | |  | | | |
| DRB1*15:01:01 | | 0.04 | |  | | |  |  | | |  | | | |
| DRB1*01:02:01 | | 0.03 | |  | | |  |  | | |  | | | |
| DRB1*01:03:01 | | 0.02 | |  | | |  |  | | |  | | | |
| DRB1*04:07:03 | | 0.02 | |  | | |  |  | | |  | | | |
| DRB1*10:01:01 | | 0.02 | |  | | |  |  | | |  | | | |
| DRB1*12:01:01 | | 0.02 | |  | | |  |  | | |  | | | |
|  |  | A* | B* | | | C* | DRB1* | DQB1* | | DPB1* | | | |  |
|  | *EHW p* | 0.0049** | 0.0000***** | | | 0.3468 | 0.0000***** | 0.0000***** | | 0.0102* | | | |  |

Supplementary table 1. Classical HLA alleles observed and Hardy-Weinberg equilibrium (EHW). Only alleles with more than 1% of frequency were represented.

| HLA-G | | | | HLA-F | | | | |
| --- | --- | --- | --- | --- | --- | --- | --- | --- |
| Alelle | | Frequency | | Alelle | | | | Frequency |
| G*01:01:01 | | 0.35 | | DQB1*03:01:01 | | | | 0.29 |
| G*01:03:01 | | 0.21 | | DQB1*04:02:01 | | | | 0.22 |
| G*01:01:02 | | 0.18 | | DQB1*03:02:01 | | | | 0.18 |
| G*01:04:01 | | 0.18 | | DQB1*05:01:01 | | | | 0.08 |
| G*01:01:03 | | 0.03 | | DQB1*03:03:02 | | | | 0.07 |
| G*01:04:04 | | 0.02 | | DQB1*02:01:01 | | | | 0.05 |
| G*01:05:01N | | 0.02 | | DQB1*06:02:01 | | | | 0.05 |
|  | |  | | DQB1*02:02:01 | | | | 0.03 |
|  | |  | | DQB1*05:03:01 | | | | 0.02 |
|  | |  | |  | | | |  |
| HLA-E | | | |  | | HLA-H | |  |
|  |  |  |  |  |  |  |  |  |
|  |  |  | | |  | | |  |
| Alelle | | Frequency | | Alelle | | | | Frequency |
| E*01:01:01 | | 0.64 | | H*01:01:01 | | | | 0.29 |
| E*01:03:02 | | 0.22 | | H*02:09 | | | | 0.2 |
| E*01:03:01 | | 0.12 | | H*DEL | | | | 0.19 |
|  | |  | | H*02:05:01 | | | | 0.16 |
|  | |  | | H*01:02:01 | | | | 0.04 |
|  | |  | | H*02:07:01 | | | | 0.03 |
|  | |  | | H*02:02:01 | | | | 0.02 |
|  | |  | |  | | | |  |
|  |  | G* | F* | | | | H* | E* |
|  | *EHW p* | 0.1265 | 0.2289 | | | | 0.0850 | 0.6775 |

Supplementary table 2. Non-Classical HLA alleles observed and Hardy-Weinberg equilibrium (EHW). Only alleles with more than 1% of frequency were represented.
